# Spontaneous regression of primary aleukemic myeloid leukemia cutis in an adult woman: A case report and review of the literature

**DOI:** 10.1016/j.jdcr.2026.04.050

**Published:** 2026-04-30

**Authors:** Brianna R. Spiegel, Sarah J. Wu, Virginia O. Volpe, Rachel Meltzer

**Affiliations:** a Boston University Chobanian & Avedisian School of Medicine, Boston, Massachusetts; b Department of Dermatology, Brigham & Women’s Hospital, Boston, Massachusetts; c Harvard Medical School, Boston, Massachusetts; d Department of Pathology, Brigham & Women’s Hospital, Boston, Massachusetts; e Department of Medicine, Brigham and Women's Hospital, Boston, Massachusetts

**Keywords:** aleukemic leukemia cutis, extramedullary disease, immunohistochemistry, leukemic infiltration of the skin, myeloid neoplasms, NPM-1 mutation, spontaneous remission

## Introduction

Aleukemic leukemia cutis (ALC) is a rare manifestation of neoplastic leukocytes and/or leukocyte precursors in the skin, preceding presence in peripheral blood or bone marrow. We describe a case of *NPM1*-mutated aleukemic myeloid leukemia cutis in an adult female presenting as asymptomatic pink nodules on the scalp, which demonstrated apparent regression following biopsy.

## Clinical presentation

**Fig 1:**
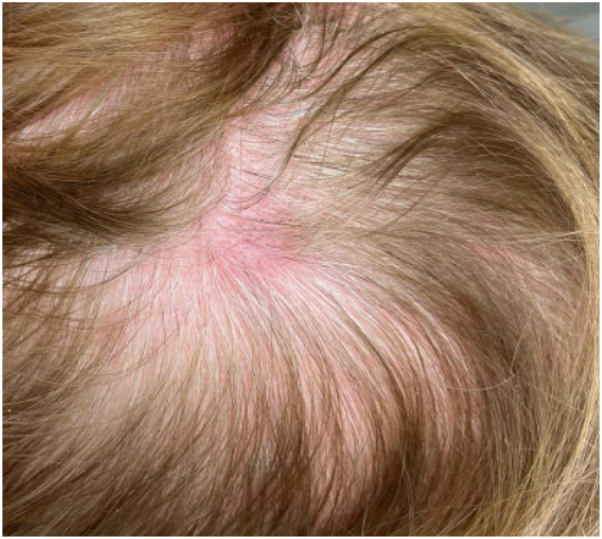
Erythematous nodule on the left crown.

**Fig 2:**
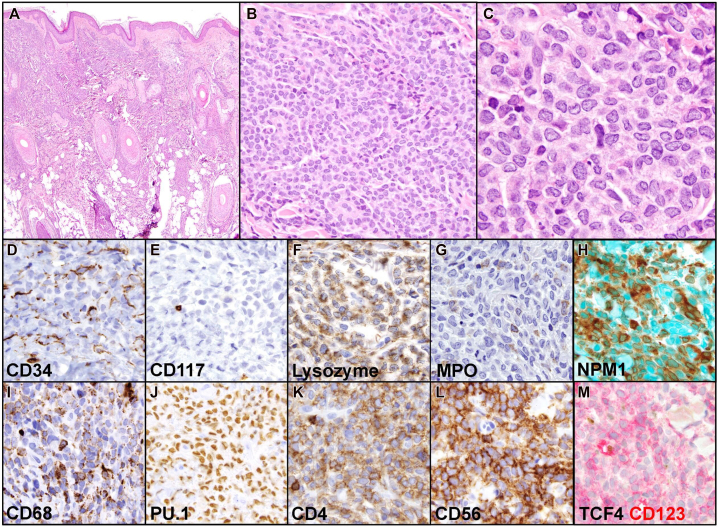
Skin punch biopsy H&E sections **(A** – 4×**, B** – 40×**, C** – 100×**)** showed involvement by acute myeloid leukemia with an immunoprofile **(D**-**M**, 100×) negative for CD34 and CD117 but positive for lysozyme, MPO (subset), cytoplasmic NPM1, CD68, PU.1, CD4, and CD56 **(D**-**L)**. A dual stain for TCF4 and CD123 **(M)** showed lesional cells positive for CD123 (*red*) and negative for TCF4 (*brown*).

An 80-year-old female with a history of nonrheumatic calcific mitral stenosis, hypertension, hypothyroidism, gout, and maternal family history of ovarian cancer presented for evaluation of 5 asymptomatic pink nodules on the scalp (Fig 1). She noticed the lesions while styling her hair about a month prior to presentation. She denied any systemic symptoms, including malaise, fevers, night sweats, fatigue, weight loss, or easy bruising or bleeding suggestive of marrow involvement. One lesion on the crown was biopsied (Fig 2). The H&E sections of the punch biopsy (Fig 2, *A*-*C*) showed skin with a superficial and deep dermal, perivascular, and interstitial atypical mononuclear infiltrate composed of medium to large sized cells with irregular, folded nuclei, open chromatin, variably prominent nucleoli, and scant to moderate amounts of pale cytoplasm. The overlying epidermis was unremarkable, demonstrating a Grenz zone.

Immunohistochemical stains (Fig 2, *D*-*M*) of atypical mononuclear cells showed positivity for NPM1 (cytoplasmic), MPO (small subset), lysozyme, CD68, CD4, CD56, and CD123. The cells were negative for TCF4, helping to exclude blastic plasmacytoid dendritic cell neoplasm.

Molecular testing performed on formalin-fixed paraffin-embedded sections of the skin punch biopsy demonstrated an *NPM1* mutation (p.W288Cfs∗12) at 31% variant allele fraction (VAF), as well as an ASXL1 mutation (p.G646Wfs∗12, 37% VAF) and 2 TET2 mutations (p.A915Ffs∗6% to 38% VAF, p.S1039Vfs∗4% to 46% VAF). A subsequent bone marrow biopsy was suboptimal for morphologic evaluation of dysplasia but did not show diagnostic morphologic features of acute leukemia, with a normal karyotype. Molecular testing demonstrated the same *ASXL1* and *TET2* mutations (approximately 12% to 14% VAF), without evidence of the *NPM1* mutation. Taken together with the skin biopsy findings, these results were consistent with involvement by *NPM1*-mutated acute myeloid leukemia (AML).

A complete blood count with differential and metabolic panel collected shortly after her initial presentation were notable for a BUN of 24 mg/dL and otherwise within normal limits. Positron emission tomography-computed tomography was negative. Initial bone marrow biopsy, aspirate, and peripheral smear performed 3 weeks after initial presentation were nondiagnostic due to inadequate sample; repeat bone marrow biopsy and aspirate at 6 weeks showed mild architectural disorganization but no diagnostic morphologic features of acute leukemia and no increase in blasts. Peripheral smear showed mature granulocytes without significant dysplasia and no circulating blasts. Flow cytometry was not performed due to inadequate blasts. All 5 lesions resolved completely without treatment approximately 1 month after biopsy and prior to initiation of systemic therapy. Approximately 3 weeks later, the patient received 7 days of azacitidine and venetoclax and elected to discontinue further therapy. She remains clinically well 16 months after initial presentation.

## Discussion

ALC is a rare manifestation of blood-borne or bone marrow–borne neoplastic leukocytes and/or leukocyte precursors in the skin prior to detection in the peripheral blood or bone marrow.^1^ ALC can present variably, including as papules, macules, plaques, nodules, ecchymoses, palpable purpura, and ulcerations.^2^ Immunohistochemical stains and molecular genetic testing is of greatest diagnostic value; expression of myeloperoxidase, lysozyme, CD68, and CD4 support a myeloid origin.^3^ ALC is associated with a poor prognosis and risk of progression to systemic leukemia, most commonly AML (approximately 13% of cases).^1^^,^^2^ The interval to progression is variable, ranging from months to several years.^4^ Combination chemotherapy, such as cytarabine and daunorubicin, has been shown to be the most effective treatment.^5^

Although apparent spontaneous regression of ALC has been reported in infants, cases in adults are rare.^6^^,^^7^ Resolution of skin nodules following biopsy has been described in 1 case, with no local recurrence or systemic progression over 7 years of follow up.^6^

In this case, the presentation of pink scalp nodules and corresponding histopathologic findings prompted consideration of a broad differential diagnosis, including pilar cysts, cylindroma, dermatofibrosarcoma protuberans, malignant proliferating trichilemmal tumor, and cutaneous B-cell lymphoma. Upon pathologic assessment, blastic plasmacytoid dendritic cell neoplasm was considered given expression of CD123, CD4 and CD56 (Fig 2, *K-M*); however, the lesion was negative for TCF4, a marker of plasmacytoid dendritic cell differentiation, which is necessarily expressed in all cases of blastic plasmacytoid dendritic cell neoplasm (Fig 2, *M*). Histiocytic sarcoma was suspected given extensive monocytic differentiation, but this feature in combination with cytoplasmic *NPM1* supported a diagnosis of extramedullary AML with monocytic differentiation.^8^ Other mutations, such as *ASXL1* and *TET2*, are also characteristic of AML; these are usually unfavorable prognostic markers.^9^ While the bone marrow biopsy was suboptimal for evaluation of dysplasia, detection of *ASXL1* and *TET2* mutations in both marrow and skin suggests a shared clonal progenitor. In the setting of normal peripheral blood counts, these mutations likely represent clonal hematopoiesis of indeterminate potential. A retrospective cohort study of patients with AML demonstrated that *NPM1* mutations can arise as a second-hit in the context of preexisting clonal hematopoiesis of indeterminate potential mutations,^10^ supporting this proposed mechanism in our case within a clone involving exclusively the skin.

This case has several limitations. Bone marrow evaluation was limited by suboptimal sampling, restricting morphologic assessment and flow cytometric analysis and may have reduced sensitivity for detecting occult systemic involvement. More comprehensive peripheral blood–based studies may have clarified whether disease was truly isolated to the skin. In addition, although the lesions resolved prior to initiation of systemic therapy, interpretation of the regression as fully spontaneous is limited by the patient’s subsequent exposure to azacitidine and venetoclax, which may have contributed to sustained remission, and by the absence of histologic confirmation of lesion resolution with repeat biopsy. Nevertheless, the diagnostic biopsy findings, including presence of a pathogenic *NPM1* mutation and supportive immunophenotype with absent TCF4 expression, strongly support cutaneous involvement by AML, and the patient remains well without recurrence or overt systemic progression during 16 months of follow-up. Given the potential for delayed progression in ALC, continued long-term clinical and hematologic surveillance remains essential.

## Conflicts of interest

None disclosed.

## References

[1] Du A.X., Hung T., Surmanowicz P., Gniadecki R. (2020). Diagnostic challenge of aleukemic leukemia cutis preceding acute myelogenous leukemia: A case report. SAGE Open Med Case Rep.

[2] Yonal I., Hindilerden F., Coskun R., Dogan O.I., Nalcaci M. (2011). Aleukemic leukemia cutis manifesting with disseminated nodular eruptions and a plaque preceding acute monocytic leukemia: a case report. Case Rep Oncol.

[3] Cibull T.L., Thomas A.B., O'Malley D.P., Billings S.D. (2007). Myeloid leukemia cutis: a histologic and immunohistochemical review. J Cutan Pathol.

[4] Wagner G., Fenchel K., Back W., Schulz A., Sachse M.M. (2012). Leukemia cutis—epidemiology, clinical presentation, and differential diagnoses. J Dtsch Dermatol Ges.

[5] Chang H., Shih L.Y., Kuo T.T. (2003). Primary aleukemic myeloid leukemia cutis treated successfully with combination chemotherapy: report of a case and review of the literature. Ann Hematol.

[6] Higaki-Mori H., Yamada N., Ozaki K., Nishimura M.F., Yoshida Y. (2025). Spontaneous complete regression of aleukaemic cutaneous myeloid sarcoma without progression to leukaemia over a long-term follow-up period. Acta Derm Venereol.

[7] Oyedepo J., Bageta M., Polubothu M. (2024). P19 spontaneous regression of aleukaemic cutis leukaemia in a male infant. Br J Dermatol.

[8] Luskin M.R., Huen A.O., Brooks S.A. (2015). NPM1 mutation is associated with leukemia cutis in acute myeloid leukemia with monocytic features. Haematologica.

[9] Padmakumar D., Chandraprabha V.R., Gopinath P. (2021). A concise review on the molecular genetics of acute myeloid leukemia. Leuk Res.

[10] Cappelli L.V., Meggendorfer M., Baer C. (2021). Indeterminate and oncogenic potential: CHIP vs CHOP mutations in AML with NPM1 alteration. Leukemia.

